# Comparison of laboratory and immediate diagnosis of coagulation for patients under oral anticoagulation therapy before dental surgery

**DOI:** 10.1186/1746-160X-1-12

**Published:** 2005-11-29

**Authors:** Birgit Kruse-Loesler, Matthias Kelker, Johannes Kleinheinz

**Affiliations:** 1 Department of Cranio-Maxillofacial Surgery, University of Muenster, Waldeyerstr. 30, D-48149 Muenster, Germany

## Abstract

**Background:**

Dental surgery can be carried out on patients under oral anticoagulation therapy by using haemostyptic measures. The aim of the study was a comparative analysis of coagulation by laboratory methods and immediate patient diagnosis on the day of the planned procedure.

**Methods:**

On the planned day of treatment, diagnoses were carried out on 298 patients for Prothrombin Time (PT), the International Normalised Ratio (INR), and Partial Thromboplastin Time (PTT). The decision to proceed with treatment was made with an INR < 4.0 according to laboratory results.

**Results:**

Planned treatment did not go ahead in 2.7% of cases. Postoperatively, 14.8% resulted in secondary bleeding, but were able to be treated as out-patients. 1.7% had to be treated as in-patients. The average error between the immediate diagnosis and the laboratory method: 95% confidence interval was -5.8 ± 15.2% for PT, -2.7 ± 17.9 s for PTT and 0.23 ± 0.80 for INR. The limits for concordance were 9.4 and -21.1% for PT, 15.2 and -20.5 s for PTT, and 1.03 and -0.57 for INR.

**Conclusion:**

This study showed a clinically acceptable concordance between laboratory and immediate diagnosis for INR. Concordance for PT and PTT did not meet clinical requirements. For patients under oral anticoagulation therapy, patient INR diagnosis enabled optimisation of the treatment procedure when planning dental surgery.

## Background

To prevent thrombosis and embolism, patients are treated increasingly as out-patients and are sometimes given anticoagulant therapy for many years. The anticoagulant with coumarin derivatives is widely distributed. As vitamin K antagonists, these derivatives inhibit the g-carboxylation of glutamic acids in the synthesis of coagulation factors II, VII, IX and X in the liver. With regards to comparability and standardisation of test results for oral anticoagulation therapy, the International Normalised Ratio (INR) is recommended by WHO for monitoring of patients' coagulation physiology. INR values between 2.0 and 3.0 are recommended for thromboembolic illnesses, atrial fibrillation, heart valve diseases and myocardial infarct. For mechanical heart valves and recurring embolism, an INR value between 2.5 and 3.5 is set.

Haemorrhages, which can occur after teeth extractions, for example, only present a significant risk for patients under anticoagulation therapy in exceptional cases [7]. In contrast, stopping anticoagulation therapy independently before dental surgery procedures can present patients with unnecessary life-threatening risks from thromboembolism [27]. With INR values for therapy between 2.0 and 3.5, extractions of one or more teeth and uncomplicated osteotomies, taking into account relevant local haemostasis methods without danger of haemorrhaging, are possible [12], whereby the INR value is to be determined pre-operatively on the day of operation [21]. For immediate patient diagnosis of the coagulation status, instruments have been developed which are used in operating theatres and in intensive care [18]. The same systems are used by patients to self-monitor oral anticoagulation [1,17]. The aim of this study was a comparative analysis of coagulation and treatment planning for immediate diagnosis of patients and diagnosis in the main laboratory.

## Methods

### Patients

A total of 298 patients who received dental treatment under anticoagulation therapy were included in this study. The period of acquisition reached from 10.10.2002 to 16.04.2004. The group tested consisted of 106 women and 192 men with an age range of 11–91 years and an average age of 60.1 years. Anticoagulation treatment was carried out after thromboembolic illnesses (30.5%), atrial fibrillation (27.4%), myocardial infarct (15.1%) and heart valve replacement (34.0%). Coumarin therapy was carried out for 92.4% of patients. 6.6% were treated under general anaesthesia and oral anticoagulation was administered by intravenous heparin. Additional inhibition of thrombocyte aggregation by acetylsalicylic acid or ticlopidine occurred for 2.7%. The decision to proceed with treatment was made with an INR < 4.0 according to laboratory results [2]. 83.0% of patients underwent surgery, where predominantly a single tooth was extracted and small osteotomies were carried out. 28.3% received preservation treatment, 9.4% prosthetic treatment and 10.4% periodontal treatment. To protect against an increased risk of intra- and postoperative complications with cardiovascular diseases, 56.7% were administered intravenously and 39.7% were put under ECG monitoring. Endocarditis prophylaxis was carried out for 43.4% in accordance with the guidelines of the German Society for Heart Diseases.

Local haemostyptic measures consisted of sealing the thick wound in a collagen dressing (66.0%), a fibrin adhesive (27.4%), and the use of a protective plate (18.9%). A local flap coverage was used in 15.1% of cases.

### Examination methods

Patients' intra- and postoperative trends were recorded and complications were documented. Before treatment, capillary blood was extracted. The diagnosis for Prothrombin Time (PT), International Normalised Ration (INR) and Partial Thromboplastin Time (PTT) was carried out with a CoaguChek Pro device (Roche Diagnostics, Mannheim, Germany). CoaguChek Pro PT cassettes contain thromboplastin from rabbit brains (ISI 2.04). As a PTT reagent, cow brain sulphatide was used as an activator, and soya phosphatide as a platelet substitute. The measuring principle of the machine is based on laser-photometric detection of erythrocyte movement, which is suspended when coagulation begins to set in. Measurement and quality control were carried out, in accordance with the manufacturer's instructions, by 3 experienced assistant medical technicians. At the same time, blood tests were taken in the coagulation tubes (0.106 mol/l citrate, 10%) and analysed in the clinic's main laboratory. Both the PT and PTT diagnoses were carried out with a BCS coagulation analyser (Dade Behring, Marburg, Germany). Thromborel S (tissue factor from a human placenta, ISI 1.09) and Pathromtin SL (Dade Behring, Marburg, Germany) were used as reagents.

### Analysis and statistics

The test results were analysed by a thorough data analysis with determination of average values and standard deviations. As a cohesive measurement between the parameters, the correlation coefficient r with the corresponding test (HO: r = 0) was used. With p values < 0.01 the correlation was accepted. Selected parameter combinations were depicted in range diagrams with the corresponding regression line. The concordance between measurement methods was analysed in accordance with *Bland and Altman *[3]. Average and relative errors and the absolute and relative limits of concordance were calculated and depicted in diagram form. When comparing methods, a relative error of ± 10% and relative limits of concordance at ± 25% were assessed to be clinically acceptable [19].

## Results

Planned treatment did not go ahead in 2.7% of cases. 2.3% also had an INR value > 4.0 in immediate diagnosis. 2.3% of immediate diagnoses were in the same measurement range; laboratory diagnoses gave an INR value > 4.0. Intra-operatively, there was the complication of one major haemorrhage. Postoperatively, 14.8% resulted in secondary bleeding, but were able to be treated as out-patients. 1.7% had to be treated as in-patients.

**Figure 1:**
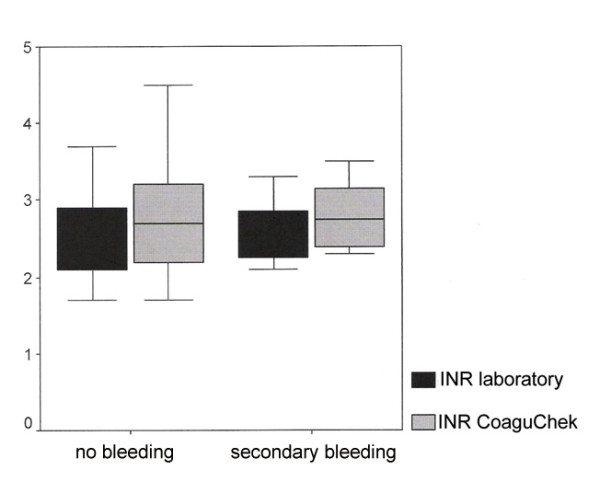
Immediate (CoaguChek) and laboratory diagnosis of INR concerning secondary bleeding.

No significant difference was determined in coagulation values for patients who did and did not suffer secondary bleeding (Fig. 1). On analysis of postoperative secondary bleeding within the category of the "additional operative measures" variable, a high proportion (33.3%) was treated with a local flap reconstruction (Tab. 1).

**Table 1:** Frequency of secondary bleeding concerning additional surgical treatment options

	no bleeding	secondary bleeding
suture	88.9%	11.1%
collagen dressing	92.3%	7.7%
fibrin adhaesive	85.0%	15.0%
local flap coverage	66.7%	33.3%

**Figure 2:**
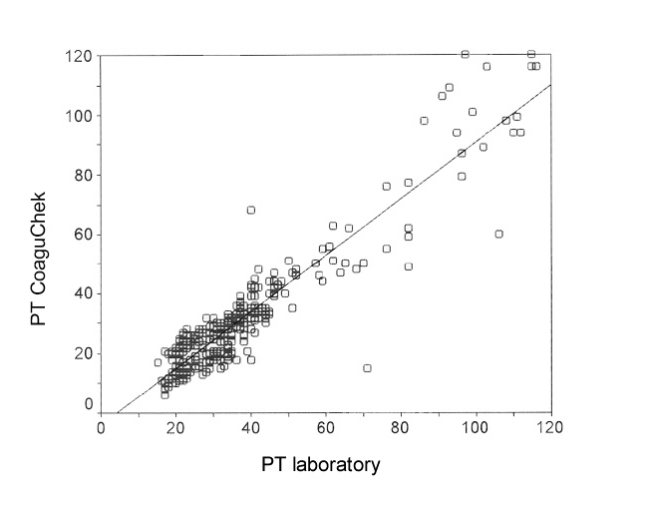
Correlation of immediate (CoaguChek) and laboratory diagnosis of Prothrombin Time (PT) [%] (Pearsons coefficient r = 0.93).

**Figure 3:**
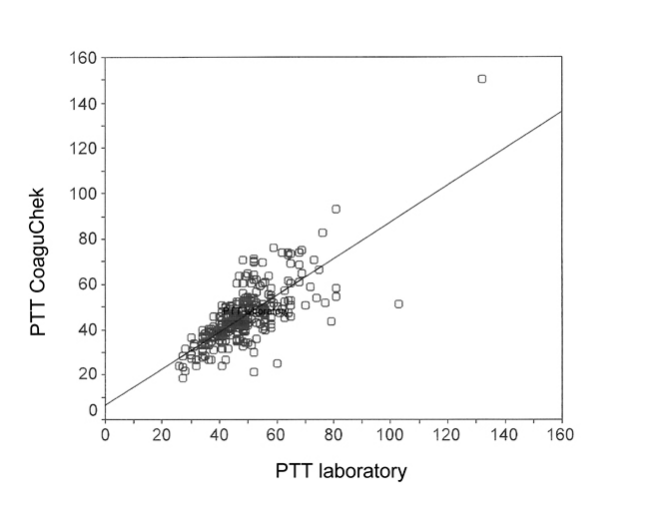
Correlation of immediate (CoaguChek) and laboratory diagnosis of Partial Thromboplastin Time (PTT) [s] (Pearsons coefficient r = 0.74).

**Figure 4:**
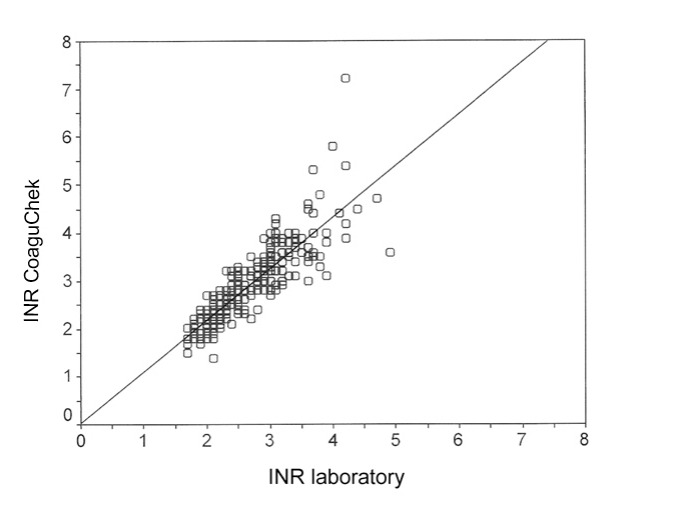
Correlation of immediate (CoaguChek) and laboratory diagnosis of the International Normalised Ratio (INR) (Pearsons coefficient r = 0.86)

**Figure 5:**
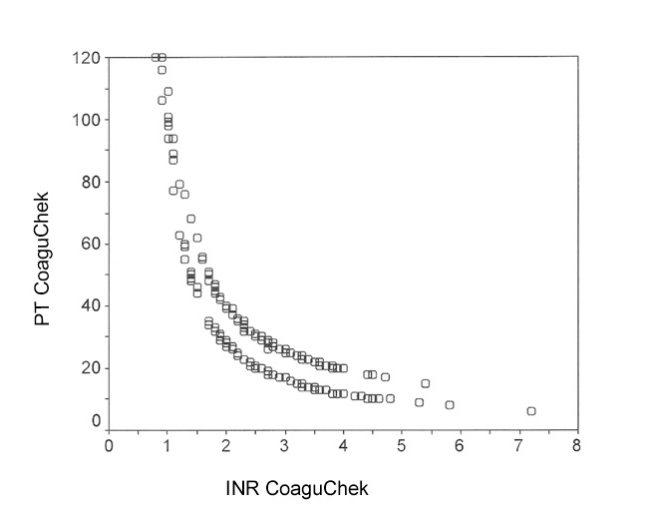
Correlation of immediate diagnosis (CoaguChek) of the Prothrombin Time (PT) [%] and the International Normalised Ratio (INR) (Spearman coefficient r = -0.89)

Fig. 2 shows the range diagram of PT diagnoses from CoaguChek and the laboratory with linear regression and confidence interval. The results for PTT and INR are shown in the same way in Fig. 3 and Fig. 4. The correlation of CoaguChek PT and INR values show a charge-dependent difference in the curve progression (Fig. 5).

**Figure 6:**
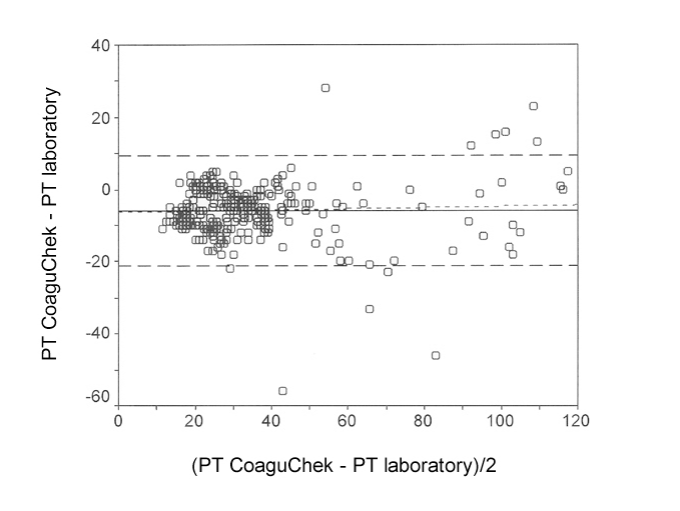
Relative error between the laboratory and immediate determination method concerning Prothrombin time (PT)

**Figure 7:**
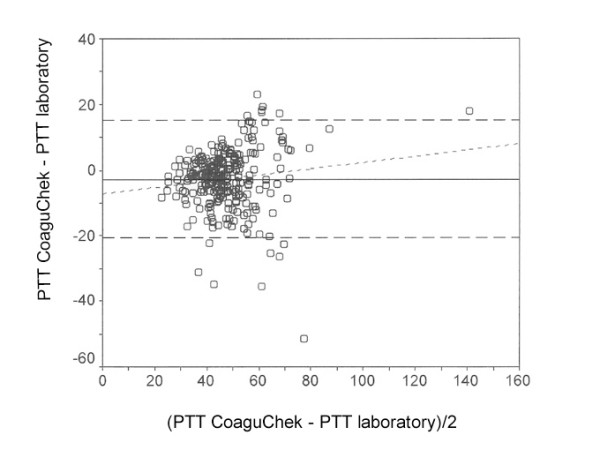
Relative error between the laboratory and immediate determination method concerning Partial Thromboplastin Time (PTT).

**Figure 8:**
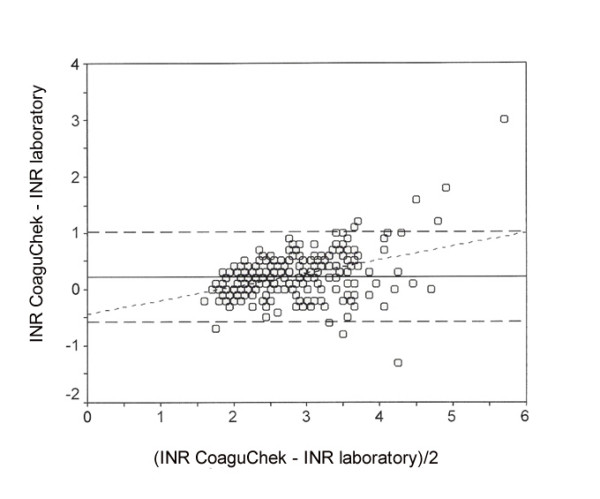
Relative error between the laboratory and immediate determination method concerning International Normalised Ratio (INR).

The relative error between the laboratory and immediate determination method ± 95% confidence interval was -5.8 ± 15.2% for PT (Fig. 6), -2.7 ± 17.9 s for PTT (Fig. 7) and 0.23 ± 0.80 for INR (Fig. 8). The limits for concordance were 9.4 and -21.1% for PT, 15.2 and -20.5 s for PTT, and 1.03 and -0.57 for INR.

The relative error between the laboratory methods and immediate diagnosis was 21.3% with an average PT of 34.5%, 6.3% with an average PTT of 47.2 s, and 7.6% with an average INR of 2.77. The relative limit for concordance between laboratory methods and immediate diagnosis was 48.6% for PT, 35.7% for PTT and 24.3% for INR.

## Discussion

The proportion of patients with postoperative secondary bleeding is comparable with the literature [2,6,16] as regards local haemostyptic measures used. Good results were established from the collagen dressing and the use of fibrin adhesive or tranexamic acid in various studies [4,6,15,22,26]. The plastic cover doubled the frequency of secondary bleeding and should therefore be reserved for the cover of maxillary sinus connection for the treatment of patients under oral anticoagulation therapy. Studies on the precision of measurement devices for patient diagnosis of the coagulation status established a firm concordance with laboratory results [5,25], whereby correlation analyses were mostly used for assessment of the concordance. Neither the determination of the correlation coefficient nor the implementation of a regression analysis are considered to be suitable measures for comparing measurement methods [3]. A lower average error with INR diagnosis with a partially more marked distribution of values was established [1,24]. Measurement devices that used thromboplastin with an ISI value > 2.0 showed a decrease in concordance with comparative tests [11,13,23].

Concordance for PTT diagnoses are also the subject of controversial discussion. There are also studies on good concordance [20], as well as studies on large deviations between laboratory and immediate diagnoses [9,18,19]. In this study, a relative error of 10% between methods was regarded as clinically acceptable for PTT and INR. The PT diagnosis was clearly above the required value, which can partly be traced back to the charge-specific differences in calibration. The relative limits of concordance at 25% could only be maintained for INR. For PT and PTT the limit was exceeded. Comparable results for INR diagnoses as regards the average error and the limits of concordance were published, whereby clear differences were given at four research centres [8]. In previous studies, a positive average error with an increase in rising INR values was established from the difference between immediate and laboratory diagnoses [14]. A short treatment time using oral anticoagulation was given as a possible cause, which must not be assumed in the current study regarding predominantly long-term treatment. There is a possible error in the use of thromboplastins of differing sensitivity and the lack of calibration of the ISI with immediate diagnosis [10].

## Conclusion

In conclusion, patient INR diagnosis with the CoaguChek Pro device allows for clinically acceptable optimisation of the treatment procedure when planning dental surgery for patients under oral anticoagulation therapy.

## Competing interests

The author(s) declare that they have no competing interests.

## Authors' contributions

BKL set up the design of the study, performed the surgical part, and helped to draft the manuscript. MK carried out the statistical analysis. JK performed the statistical analysis and participated in the design of the study, the coordination of the patients and helped to draft the manuscript. All authors read and approved the final version of the manuscript.
